# Supplementary material for: Modeling glioblastoma heterogeneity as a dynamic network of cell states
Source: Mol Syst Biol. 2021 Sep 16;17(9):e10105. doi: 10.15252/msb.202010105 (PMC8444284; doi:10.15252/msb.202010105)
Supplement: Supplementary file 5 — Source Data for Figure 3 [file MSB-17-e10105-s001.zip › Figure3A_sourcedata/GSEA_3065/hallmarks_state5.GseaPreranked.1623416302988/HALLMARK_COAGULATION.html]

Details for gene set HALLMARK\_COAGULATION[GSEA]

|  || Dataset | state5 |
| Phenotype | NoPhenotypeAvailable |
| Upregulated in class | na\_pos |
| GeneSet | HALLMARK\_COAGULATION |
| Enrichment Score (ES) | 0.32021567 |
| Normalized Enrichment Score (NES) | 1.1664767 |
| Nominal p-value | 0.1747212 |
| FDR q-value | 0.25932452 |
| FWER p-Value | 0.861 |
Table: GSEA Results Summary

  

Fig 1: Enrichment plot: HALLMARK\_COAGULATION      
 Profile of the Running ES Score & Positions of GeneSet Members on the Rank Ordered List

  

| PROBE | GENE SYMBOL | GENE\_TITLE | RANK IN GENE LIST | RANK METRIC SCORE | RUNNING ES | CORE ENRICHMENT || 1 | CD9 |  |  | 14 | 0.857 | 0.0586 | Yes |
| 2 | PLAT |  |  | 37 | 0.714 | 0.1063 | Yes |
| 3 | LAMP2 |  |  | 69 | 0.584 | 0.1439 | Yes |
| 4 | TIMP1 |  |  | 109 | 0.518 | 0.1759 | Yes |
| 5 | TFPI2 |  |  | 124 | 0.504 | 0.2097 | Yes |
| 6 | CLU |  |  | 162 | 0.474 | 0.2389 | Yes |
| 7 | CTSV |  |  | 193 | 0.443 | 0.2666 | Yes |
| 8 | LRP1 |  |  | 259 | 0.404 | 0.2877 | Yes |
| 9 | SPARC |  |  | 327 | 0.370 | 0.3062 | Yes |
| 10 | ADAM9 |  |  | 421 | 0.334 | 0.3193 | Yes |
| 11 | HTRA1 |  |  | 690 | 0.266 | 0.3080 | Yes |
| 12 | PRSS23 |  |  | 831 | 0.235 | 0.3088 | Yes |
| 13 | CTSB |  |  | 902 | 0.225 | 0.3168 | Yes |
| 14 | TIMP3 |  |  | 1191 | 0.186 | 0.2977 | Yes |
| 15 | PROS1 |  |  | 1210 | 0.183 | 0.3085 | Yes |
| 16 | SERPINE1 |  |  | 1221 | 0.182 | 0.3202 | Yes |
| 17 | FN1 |  |  | 1342 | 0.167 | 0.3185 | No |
| 18 | BMP1 |  |  | 1550 | 0.147 | 0.3057 | No |
| 19 | C1R |  |  | 1625 | 0.140 | 0.3072 | No |
| 20 | FBN1 |  |  | 1756 | 0.129 | 0.3017 | No |
| 21 | MMP11 |  |  | 1813 | 0.126 | 0.3043 | No |
| 22 | MMP2 |  |  | 1824 | 0.125 | 0.3120 | No |
| 23 | MMP15 |  |  | 1942 | 0.116 | 0.3070 | No |
| 24 | LGMN |  |  | 2091 | 0.106 | 0.2979 | No |
| 25 | CAPN5 |  |  | 2765 | 0.065 | 0.2271 | No |
| 26 | PLAU |  |  | 3407 | 0.031 | 0.1575 | No |
| 27 | CPQ |  |  | 3428 | 0.031 | 0.1574 | No |
| 28 | SIRT2 |  |  | 3589 | 0.024 | 0.1411 | No |
| 29 | CTSO |  |  | 3624 | 0.023 | 0.1389 | No |
| 30 | FURIN |  |  | 3719 | 0.019 | 0.1298 | No |
| 31 | THBS1 |  |  | 3755 | 0.017 | 0.1271 | No |
| 32 | LTA4H |  |  | 4159 | 0.000 | 0.0820 | No |
| 33 | SH2B2 |  |  | 5009 | -0.033 | -0.0107 | No |
| 34 | PREP |  |  | 6101 | -0.080 | -0.1273 | No |
| 35 | CSRP1 |  |  | 6689 | -0.113 | -0.1850 | No |
| 36 | FYN |  |  | 6823 | -0.123 | -0.1913 | No |
| 37 | RABIF |  |  | 6945 | -0.132 | -0.1956 | No |
| 38 | USP11 |  |  | 7153 | -0.151 | -0.2082 | No |
| 39 | DUSP6 |  |  | 7258 | -0.162 | -0.2084 | No |
| 40 | PEF1 |  |  | 7425 | -0.184 | -0.2140 | No |
| 41 | DUSP14 |  |  | 7487 | -0.190 | -0.2076 | No |
| 42 | GSN |  |  | 7578 | -0.201 | -0.2035 | No |
| 43 | CRIP2 |  |  | 7935 | -0.263 | -0.2249 | No |
| 44 | CAPN2 |  |  | 8080 | -0.293 | -0.2205 | No |
| 45 | ISCU |  |  | 8183 | -0.321 | -0.2093 | No |
| 46 | MSRB2 |  |  | 8252 | -0.340 | -0.1931 | No |
| 47 | GNG12 |  |  | 8328 | -0.362 | -0.1760 | No |
| 48 | WDR1 |  |  | 8554 | -0.463 | -0.1687 | No |
| 49 | ARF4 |  |  | 8567 | -0.467 | -0.1373 | No |
| 50 | ANXA1 |  |  | 8595 | -0.484 | -0.1063 | No |
| 51 | GNB2 |  |  | 8790 | -0.644 | -0.0828 | No |
| 52 | S100A13 |  |  | 8820 | -0.670 | -0.0390 | No |
| 53 | RAC1 |  |  | 8919 | -0.815 | 0.0073 | No |
Table: GSEA details [plain text format]

  

Fig 2: HALLMARK\_COAGULATION: Random ES distribution      
 Gene set null distribution of ES for **HALLMARK\_COAGULATION**
